# Metabolomics of testosterone enanthate administration during severe-energy deficit

**DOI:** 10.1007/s11306-022-01955-y

**Published:** 2022-11-30

**Authors:** Jesse A. Stein, J. Philip Karl, Claire E. Berryman, Melissa N. Harris, Jennifer C. Rood, Stefan M. Pasiakos, Harris R. Lieberman

**Affiliations:** 1 Military Nutrition Division, US Army Research Institute of Environmental Medicine (USARIEM), Natick, MA USA; 2 Department of Nutrition and Integrative Physiology, Florida State University, Tallahassee, FL USA; 3 Louisiana State University’s Pennington Biomedical Research Center, Baton Rouge, LA USA

**Keywords:** Semi-starvation, Sport metabolomics, Anabolic, Military stress

## Abstract

**Introduction:**

Testosterone administration attenuates reductions in total body mass and lean mass during severe energy deficit (SED).

**Objectives:**

This study examined the effects of testosterone administration on the serum metabolome during SED.

**Methods:**

In a double-blind, placebo-controlled clinical trial, non-obese men were randomized to receive 200-mg testosterone enanthate/wk (TEST) (n = 24) or placebo (PLA) (n = 26) during a 28-d inpatient, severe exercise- and diet-induced energy deficit. This study consisted of three consecutive phases. Participants were free-living and provided a eucaloric diet for 14-d during Phase 1. During Phase 2, participants were admitted to an inpatient unit, randomized to receive testosterone or placebo, and underwent SED for 28-d. During Phase 3, participants returned to their pre-study diet and physical activity habits. Untargeted metabolite profiling was conducted on serum samples collected during each phase. Body composition was measured using dual-energy X-ray absorptiometry after 11-d of Phase 1 and after 25-d of Phase 2 to determine changes in fat and lean mass.

**Results:**

TEST had higher (Benjamini–Hochberg adjusted, q < 0.05) androgenic steroid and acylcarnitine, and lower (q < 0.05) amino acid metabolites after SED compared to PLA. Metabolomic differences were reversed by Phase 3. Changes in lean mass were associated (Bonferroni-adjusted, p < 0.05) with changes in androgenic steroid metabolites (r = 0.42–0.70), acylcarnitines (r = 0.37–0.44), and amino acid metabolites (r = − 0.36–− 0.37). Changes in fat mass were associated (p < 0.05) with changes in acylcarnitines (r = − 0.46–− 0.49) and changes in urea cycle metabolites (r = 0.60–0.62).

**Conclusion:**

Testosterone administration altered androgenic steroid, acylcarnitine, and amino acid metabolites, which were associated with changes in body composition during SED.

**Supplementary Information:**

The online version contains supplementary material available at 10.1007/s11306-022-01955-y.

## Introduction

Severe energy deficit (~ 50–100% total daily energy expenditure) is a potent physiological stress commonly encountered in physically demanding occupations (i.e., military personnel, wildland firefighters), competitive weight-sport athletes (i.e., wrestling, body building), and during restricted eating that initiates a series of physiological perturbations similar to starvation (Karl et al., 2017; Monteleone et al., 2021; Rossow et al., 2013; Lieberman et al., 2017; Montain et al., 2008; Cahill, 2006; Murphy et al., 2018; Tassone & Baker, 2017). Anabolic hormones including insulin and testosterone decline during severe energy deficit while glucocorticoids (i.e. cortisol) increase (Friedl et al., 2000; Pasiakos et.al, 2019). As a result, energy deficits can induce a hypogonadal state that increases the use of fatty acids and protein stores (sequestered from skeletal muscle) for energy production (Church et al., 2019). Ultimately, if severe or prolonged, reliance on protein and fat stores for energy contributes to decrements in total body mass and fat-free mass that results in reduced muscular strength and exercise tolerance (Braun & Marks, 2015; Karl et al., 2017; Murphy et al., 2018; Rossow et al., 2013). For military personnel, these physical functions are essential because lower-body muscular power and exercise tolerance are required for many critical tasks, including performance during direct-fire engagements that increase soldier survivability (Stein et al., 2021, Stein et al., 2022).

High-protein diets have been used to improve skeletal muscle anabolism during eucaloric feeding and preserve lean mass during hypocaloric feeding (Morales et al., 2017; Pasiakos et al., 2015). However, these diets lack efficacy during the severe energy deficits often experienced by military personnel (Margolis et al., 2014, 2016; Berryman et al., 2018), and alternative solutions are needed. Testosterone administration is one potential solution as the hormone acts in a dose-dependent fashion to increase anabolic balance, lean muscle mass, and muscular strength in healthy men (Bhasin et al., 1996, 2001). However, whether testosterone’s anabolic effects persist during severe energy deficit, and if so, the extent to which changes in substrate utilization are the basis for testosterone effects, is unclear. To address this gap, this study characterized the effects of exogenous testosterone using a comprehensive, untargeted metabolomic analysis during a tightly controlled diet and exercise regimen designed to induce severe energy deficit. This approach allowed for examination of multiple metabolic pathways which is not practical using conventional laboratory methods due to the very large number of metabolites detected in a single sample.

Various untargeted metabolomics approaches have been used in human studies to identify unique biochemical responses to prolonged steady-state exercise, physically demanding training programs, caloric restriction, and extreme environments (Blackburn et al., 2020, Howe et al., 2018, Karl et al., 2017, Miyata et al., 2021, Margolis et al., 2021). These studies, which may have induced an energy deficit to some degree, found perturbations in metabolomic signatures that included increased free fatty acids, acylcarnitines, omega-fatty acid intermediates, and amino acids (Blackburn et al., 2020, Howe et al., 2018, Karl et al., 2017). However, to the best of our knowledge the metabolomic response to testosterone administration during exercise- and diet-induced energy deficit has not been studied. Only one study, to our knowledge, has evaluated the effect of testosterone on the serum metabolome. The study, conducted in hypogonadal (i.e. castration) Wistar rats (Monnerat et al., 2018), demonstrated hypogonadism resulted in significant alterations in many fatty acid (e.g., long- and medium-chain fatty acids, acylcarnitines, linoleic acids) and amino acid metabolites (e.g., leucine, tryptophan, lysine) that were reversed with testosterone administration. Total body mass was not affected by testosterone treatment, but body composition was not measured, and whether lean mass was maintained or improved is unclear. Thus, the effects of testosterone on the serum metabolome, and how those effects relate to the anabolic effects of testosterone during severe energy deficit are unclear.

Our group recently conducted a study to determine the efficacy of supplemental testosterone for maintaining lean mass during a 28-d period of severe energy deficit in healthy men (Paskiakos et al., 2019). The study findings indicated testosterone supplementation during severe energy deficit: increases lean body mass, androgen receptor expression, and circulating erythropoietin; attenuates decrements in hemoglobin and hematocrit; and prevent energy deficit-induced increases in circulating ghrelin (Hennigar et al., 2020; Howard et al., 2020; Karl et al., 2020; Pasiakos et al., 2019). In this report, we address a secondary objective of that investigation: determine the effects of testosterone administration during severe energy deficit on the serum metabolome, and to identify relationships between those effects and testosterone-induced improvements in body composition.

## Materials and methods

### Participants

Physically active men (18–39 y) free of cardiometabolic disorders and with normal testosterone concentrations (total testosterone 10.4–34.7 nmol/L) were recruited for the study between April 12, 2016 and September 15, 2017. Inclusion and exclusion criteria have been previously reported (Pasiakos et al., 2017 and 2019). All participants met age-specific US Army body composition standards. Pennington Biomedical Research Center Institutional Review Board and the Human Research Protection Office of the US Army Medical Research and Material Command approved study procedures. Participants provided written informed consent prior to participation. The study was registered at www.clinicaltrials.gov (NCT02734238).

### Study design

A between-subjects, double-blind, randomized, placebo-controlled design was employed to determine the effects of testosterone on the serum metabolic profile of men undergoing severe energy deficit. Permuted-block method (4/block) and age stratification (< 29 or ≥ 29 y) were used to allocate participants. This study consisted of three phases. Phase 1 was 14-d where participants were free-living and adhering to a provided, eucaloric diet. Participants maintained normal physical activity, determined by pre-study questionnaires, during Phase 1. During Phase 2, participants were admitted to the Pennington Biomedical Research Center, randomized to receive testosterone or placebo, and experienced to severe-energy deficit (55% energy deficit) for 28-d. During Phase 2, participants were not intentionally put under psychological duress or restricted from sleep. Upon completion of Phase 2, participants began Phase 3 and were released from the inpatient unit and instructed to return to their pre-study diet and physical activity habits. Neither testosterone nor placebo were administered during Phase 3. Phase 3 ended when participants were within ± 2.5% of their baseline total body mass or on day 42 of Phase 3, whichever came first. The metabolomics outcomes presented here were measured on day 14 of the free-living, controlled feeding (CON), day 14 (SED14) and day 28 (SED28) of severe energy deficit, after 14-d of free-living, ad libitum feeding (FL), and at weight-regain (WR) time points. The body composition measures were taken on day 11 of controlled-feeding (Phase 1) and day 25 of the severe energy-deficit phase (Phase 2).

### Exercise prescription during severe energy deficit

Exercise-induced energy expenditure was increased 50% above Phase 1 total daily energy expenditure. Total daily energy expenditure was determined using a combination of the Mifflin St. Jeor Equation (activity factor set to 1.3) and physical activity screening measures (i.e., 7-d accelerometer data, 3-d activity logs)(Mifflin et al., 1990). Phase 2 exercise consisted of varied low-, moderate-, and high-intensity (40–85% VO_2peak_) aerobic exercise. Aerobic capacity (VO_2peak_) was determined during Phase 1 using a treadmill graded exercise test until volitional exhaustion. Pulmonary VO_2_ was assessed using indirect calorimetry (ParvoMedics TruOne 2400, East Sandy, UT).

Exercise prescription included walking and running (outdoors, treadmill), elliptical, stationary cycling, and walking with a weighted backpack (30% of body mass). Light calisthenics were also incorporated into the exercise prescription every 3–4 days. On average, participants performed 3.5 exercise sessions per day supervised by research staff. Exercise prescription was verified biweekly and adjusted to achieve the desired exercise-induced energy expenditure as determined by indirect calorimetry. As previously reported by Pasiakos et al. (2019), there were no differences in the absolute or relative energy deficit between groups during Phase 2. Participants were instructed to return to their pre-study physical activity routines during Phase 3.

### Dietary intake

**Fig. 1:**
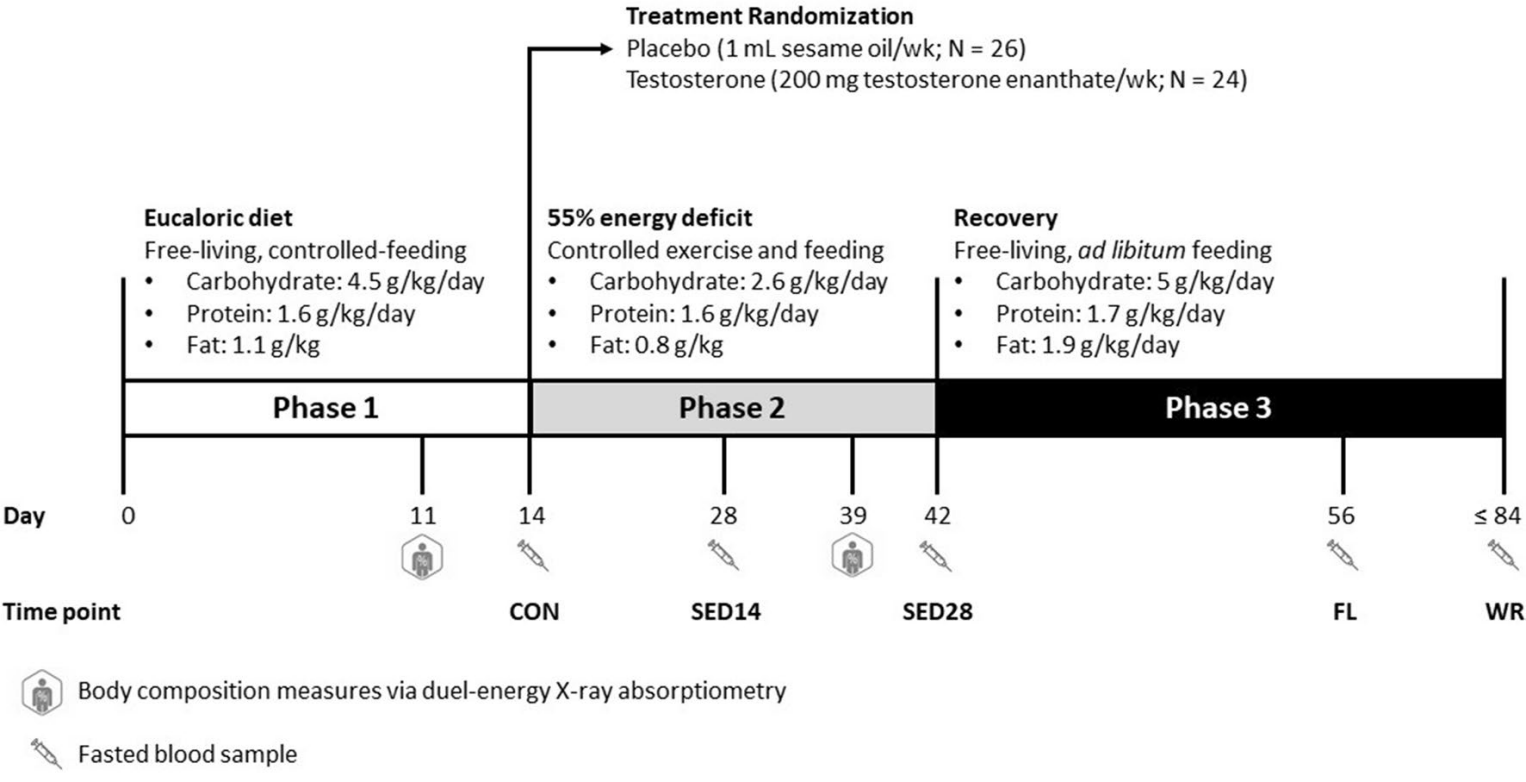
Experimental design transformed from Pasiakos et al., 2017

The Phase 1 diet was provided based on participant total daily energy expenditure. Diets provided 1.6 ± 0.2 g protein/kg/d, 30% of total energy requirements from fat, with the remaining energy from carbohydrates. Macronutrient intake is provided in Fig. 1 as reported previously (Pasiakos et al., 2019). Diet adherence was verified by research dietitians who ensured that body mass was maintained within ± 2% of baseline. Macronutrient content and energy intake were not different between groups during Phase 1.

All Phase 2 meals were provided, consumed under supervision, and contained the same macronutrient composition as Phase 1. Energy intake was restricted to 45% of the elevated total daily energy expenditure to induce a 55% energy deficit. Energy and macronutrient content were not different between groups during Phase 2 except relative protein intake was slightly higher in the placebo group (1.70 g/kg/d ± 0.06) compared to the testosterone group (1.66 ± 0.04, p < 0.05). The macronutrient composition of participant’s diet was not controlled during Phase 3. Dietary recalls were taken at FL and demonstrated there were no differences in energy or macronutrient intake between groups. Adherence to the diet and exercise prescription was ≥ 89% during the severe energy deficit protocol (Pasiakos et al., 2019).

### Testosterone administration

During Phase 2, participants received weekly intramuscular injections (4 total) of either 200 mg of testosterone enanthate in 1 mL sesame oil or placebo (1 mL sesame oil). The severe energy deficit induced was expected to result in a hypogonadal state and the dose of 200 mg of testosterone enanthate per week was selected to maintain a eugonadal testosterone level during Phase 2 (Bhasin et al., 2001; Fernández-Balsells et al., 2010).

### Body composition

Body composition was measured after an overnight fast and morning void by dual-energy X-ray absorptiometry (Lunar iDXA, GE Healthcare, Madison, WI). Lean mass was the total body mass minus fat mass and bone mineral content as previously described by Pasiakos et al. (2019).

### Plasma testosterone concentrations

Total testosterone concentrations were determined as previously reported (Pasiakos et al., 2019). Briefly, fasted blood samples were collected at baseline, CON, SED14, SED28, FL, and WR between 6:00AM and 9:00 AM to control for the effects of circadian variation in total testosterone concentrations (Siemens Immulite 2000, Llanberis, UK).

### Serum metabolomics

Fasted blood samples were collected to assess global metabolite profiles in serum. All samples were collected between 6:00 AM and 9:00 AM to minimize effects of circadian variations (Minami et al., 2009). Samples were processed immediately, and serum was isolated and stored at − 80 °C until being shipped to Metabolon, Inc. (Durham, NC) for global metabolomic profiling. Prior to the first step of extraction protocol, several recovery standards were added and analyzed with experimental samples for quality control. Samples were prepared for analysis using the automated MicroLab Star system (Hamilton Company). Each sample was divided into separate aliquots and analyzed using four separate methods: two separate reverse phase/ultra-phase liquid chromatograph-tandem mass spectrometry with positive ion electrospray ionization, one reverse phase/ultra-phase liquid chromatography-tandem mass spectrometry with negative ion electrospray ionization, and one high-performance liquid chromatography with negative ion electrospray ionization.

All methods utilized ACQUITY ultra-performance liquid chromatography (Waters, Milford, MA) and a high resolution/accurate mass spectrometer (Thermo Scientific, Hudson, NH) that interfaced with a heated electrospray ionization source and Orbitrap mass analyzer operating at 35,000 mass resolution. Sample extracts were dried and reconstituted in compatible solvents appropriate for the method used. To ensure injection and chromagraphic consistency, each reconstituting solvent contained a series of standards at fixed concentration. To chromatographically optimize for hydrophilic compounds, one aliquot was analyzed in acidic positive ion conditions by gradient eluting the extract from a C18 column (Waters UPLC BEH C18-2.1 × 100 mm, 1.7 µm) with water and methanol, containing 0.05% perfluoropentanoic acid (PFPA) and 0.1% formic acid (FA). To chromatographically optimize for hydrophobic compounds, another aliquot was analyzed using acidic positive ion conditions by gradient eluting from the same C18 column using methanol, acetonitrile, water, 0.05% PFPA and 0.01% FA and at a higher organic content. An additional aliquot was analyzed using basic negative ion optimized conditions using a separate C18 column. The basic extracts were gradient-eluted from the column using methanol and water, however with 6.5 mM ammonium bicarbonate (pH = 8). The final aliquot was analyzed via negative ionization following elution from a hydrophilic interaction chromagraphic column (Waters UPLC BEH amide 2.1 × 150 mm, 1.7 µm) using a gradient consisting of water and acetonitrile with 10 mM ammonium formate, pH 10.8. The mass spectrometry analysis alternated between mass spectrometry and data-dependent MS^n^ scans using dynamic exclusion and the scan range covered 70–1000 mass to charge ratio (m/z).

Raw data were extracted, peak-identified and quality control processed using Metabolon, Inc.’s hardware and software. Compounds were identified by comparison to library entries of purified standards or recurrent unknown entities. Biochemical identifications were based on retention index within a narrow retention index window of the proposed identification, accurate mass match to the library ± 10 ppm, and the tandem-mass spectrometry forward and reverse (MS/MS) scores between the experimental data and authentic standards. The MS/MS scores were based on a comparison of the ions present in the experimental spectrum to the ions present in the library spectrum. Samples were quality controlled and curated using software developed at Metabolon, Inc. to ensure that high quality data were used for statistical analyses.

### Statistical analysis

Metabolon, Inc. provided batch-normalized data for each sample. Batch-normalization is conducted for analyses spanning multiple days, and corrects for variation resulting from inter-day instrument variability. Batch-normalization values were determined by dividing each sample’s raw peak intensity (i.e. area under the curve) values by the median of the samples in each instrument batch. Batch normalized data for 958 metabolites was uploaded to MetaboAnalyst (Version 5.0) for analysis (Pang et al., 2021). Metabolites were subsequently removed if not detected in > 20% of samples and any remaining missing data were imputed using 1/5 of the minimum value for that feature across all samples. Metabolites with nearly constant values were then detected and removed using robust estimate of interquantile ranges. Finally, to better approximate a normal distribution, data were generalized logarithm transformed and auto scaled (mean-centered and divided by the standard deviation of each variable) for statistical analysis.

Principle component analysis (PCA) was used to visualize serum metabolomic signatures over time and between groups. PCA was followed with Orthogonal PLS-DA when differences between groups were identified. Linear mixed models with subject included as a random effect, and group, time and the group-by-time interaction as fixed factors was used to identify differences in the trajectories of individual metabolites over time between testosterone *versus* placebo. False discovery rate (FDR) correction of p-values was completed using the Benjamini–Hochberg method. If a significant interaction was detected (FDR < 0.05), pairwise comparisons were conducted to identify differences for each metabolite within and between groups. Significant interactions were visualized using a heatmap with hierarchical Ward’s clustering with the Euclidean distance. P-values for pairwise comparisons were adjusted using a Bonferroni correction. Associations between changes in metabolite levels from CON to SED28 and changes in lean and fat mass over the same time period were assessed by Pearson’s product-moment correlations with p-values adjusted using the Benjamini–Hochberg method. Change scores were also used to determine the relationship between changes in androgenic steroid metabolites and changes in other metabolites where significant interactions were found. Significance was set at p < 0.05 and FDR < 0.05. Analyses were conducted in Metaboanalyst v.5.0, SPSS v.26 and R v.4.0.3.

## Results

**Fig. 2:**
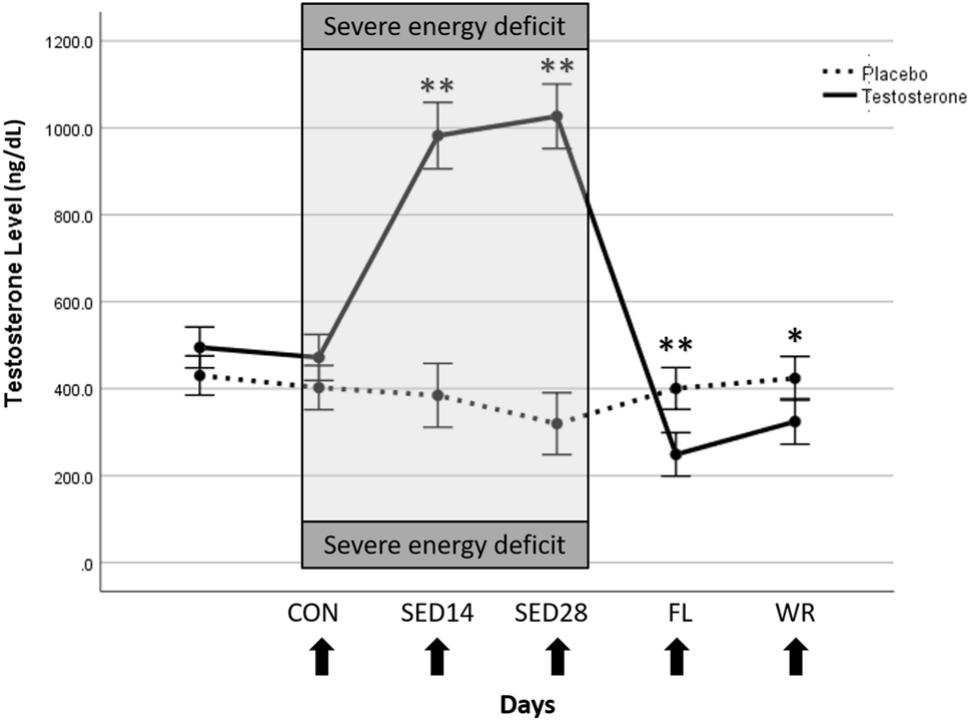
Total testosterone concentrations between testosterone and placebo conditions. * p < 0.01, **p < 0.001. Time points represent after 14-d of the free-living, controlled feeding (CON), 14- (SED14) and day 28-d (SED28) of severe energy deficit, 14-d of free-living, ad libitum feeding (FL), and weight-regain (WR)

As previously reported (Pasiakos et al., 2019), total and free testosterone were greater (p < 0.001) in the testosterone group than the placebo group during Phase 2 (Fig. 2). Additionally, during Phase 2, both testosterone and placebo groups experienced decreases in fat mass (p < 0.0001), but the testosterone group experienced an increase in lean mass that was significantly higher than changes in lean mass within the placebo group (p = 0.003; Table 1).

**Table 1:** Body composition outcomes for placebo and testosterone group

	Placebo (N = 26)			Testosterone (N = 24)			p-value		
	Phase 1	Phase 2	Phase 3	Phase 1	Phase 2	Phase 3	Phase	Treatment	Phase x Treatment
Total Body Mass	76.3 ± 10.2^bc^	71.3 ± 9.7^ac*^	74.9 ± 9.0^ab^	80.2 ± 13.5^b^	78.0 ± 12.3^ac*^	81.0 ± 12.6^b^	< 0.0001	0.086	< 0.0001
Lean Mass	56.1 ± 5.1^c^	55.8 ± 5.0^c*^	57.7 ± 5.1^ab*^	60.3 ± 10.1^bc^	62.8 ± 9.5^ac*^	64.0 ± 10.2^ab*^	< 0.0001	0.011	< 0.0001
Fat Mass	17.0 ± 6.6^bc^	12.3 ± 6.0^ac^	14.0 ± 5.4^ab^	16.6 ± 6.9^bc^	11.8 ± 6.1^ac^	13.7 ± 5.3^bc^	< 0.0001	0.817	0.926

All outcomes values represent mean ± standard deviation in kilograms. Significance was set to p < 0.05. ^a^Different from Phase 1. ^b^Different from Phase 2. ^c^Different from Phase 3. *Different between treatment groups

Serum samples for metabolomics analysis were available from all but two participants in the testosterone group during FL leaving 248 samples for analysis. Following data processing 600 compounds remained for statistical analysis.

**Fig. 3:**
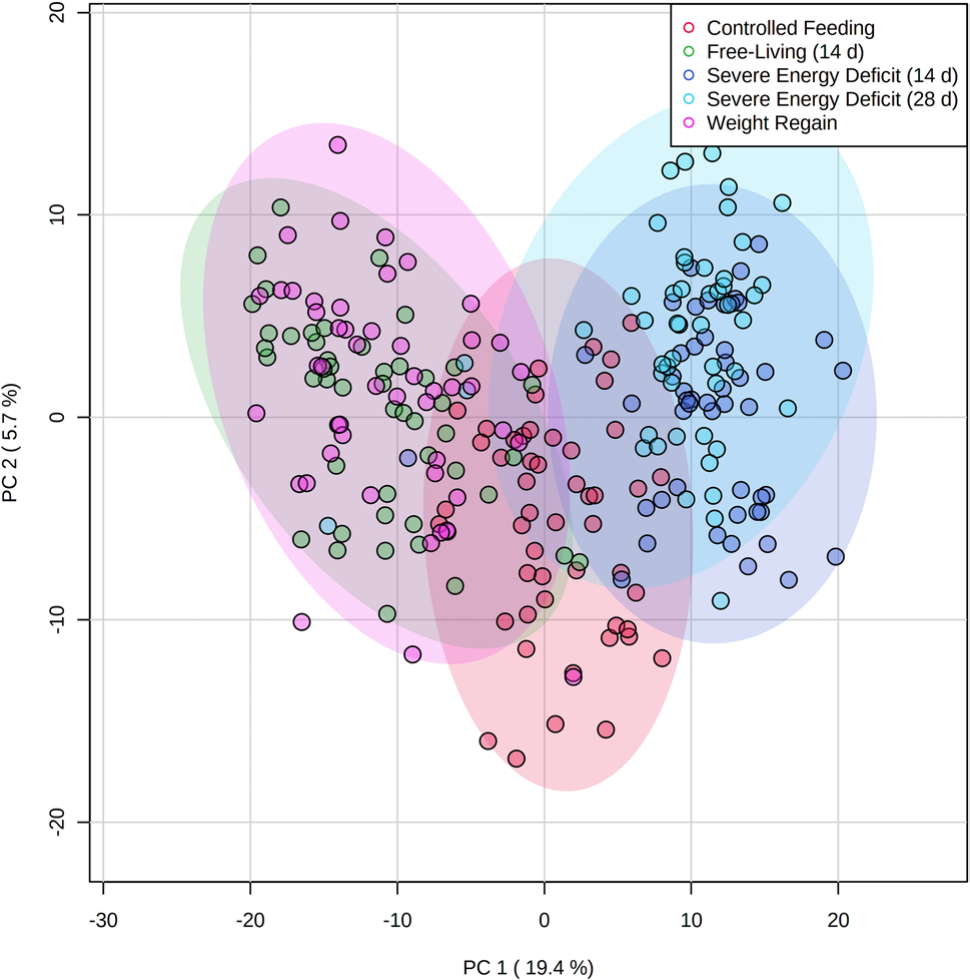
PCA scores plot during controlled feeding (Phase 1), after 14- and 28-days of severe energy deficit (Phase 2), and after 14-days of free-living and weight regain (Phase 3)

**Fig. 4:**
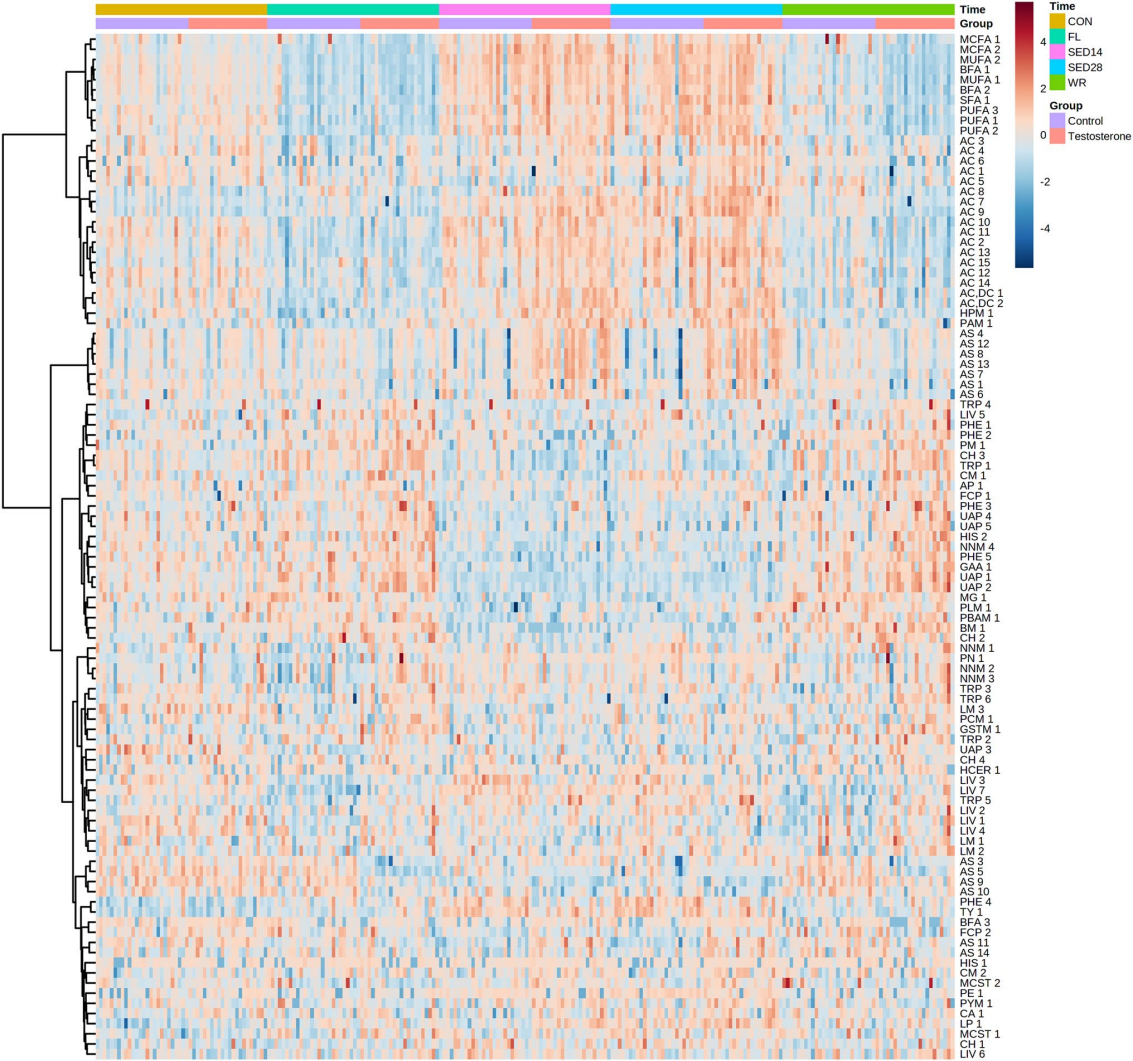
Heatmap of significant group x time interactions in response to severe energy deficit and testosterone administration. Data were clustered by Ward’s clustering with the Euclidean distance. Time points represent after 14-d of the free-living, controlled feeding (CON), 14- (SED14) and day 28-d (SED28) of severe energy deficit, 14-d of free-living, ad libitum feeding (FL), and weight-regain (WR). *AC* acylcarnitine metabolites, *AC,DC* acylcarnitine, dicarboxylate metabolites, *AP* Acetylated Peptides, *AS* Androgenic Steroids, *BFA* branch fatty acid, *BM* Benzoate metabolites, *CA* Ceramides, *CH* Chemical, *CM* creatine metabolites, *FCP* Food Component/Plant, *GAA* Gamma-glutamyl Amino Acid, *GSTM* Glycine, Serine and Threonine metabolites, *HCER* Hexosylceramides, *HIS* Histidine metabolites, *HPM* Hemoglobin and Porphyrin metabolites, *LIV* Leucine, Isoleucine and Valine metabolites, *LM* Lysine metabolites, *LP* Lysophospholipid, *MCFA* Medium Chain Fatty Acid, *MCST* Methionine, Cysteine, SAM and Taurine metabolites, *MG* Monoacylglycerol, *MUFA* Long Chain Monounsaturated Fatty Acid, *NNM* Nicotinate and Nicotinamide metabolites, *PAM* Polyamine metabolites, *PBAM* Primary Bile Acid metabolites, *PCM* Partially Characterized Molecules, *PE* Phosphatidylethanolamine, *PHE* Phenylalanine metabolites, *PLM* Phospholipid metabolites, *PM* Pentose metabolites, *PN* Vitamin B6 metabolites, *PUFA* Long Chain Polyunsaturated Fatty Acid, *PYM* Pyrimidine metabolites, *SFA* Long Chain Saturated Fatty Acid, *TRP* Tryptophan metabolites, *TY* Tyrosine metabolites, *UAP* Urea cycle; Arginine and Proline metabolites

PCA analysis revealed a clear separation by time points (Fig. 3) with PC1 and PC2 accounting for 25.1% of the variability. PC1, which accounts for the largest variation in the data, showed clear separation by time. PCA analysis showed some separation by group during SED14; however, orthogonal PLS-DA at SED14 was not significant (1000 permutations, R^2^ = 0.83, p = 0.27). Significant group x time interactions (FDR < 0.05) were identified for 102 analytes (Fig. 4, Supplement 1) that were predominately metabolites of androgenic steroid (N = 14, Supplement 2), fatty acid (N = 28, Supplement 3), and amino acid (N = 23, Supplement 5) metabolism.

Androgenic steroid metabolites responded differently across treatment conditions (Fig. 4, Supplement 2). During the severe energy deficit phase, serum androgenic steroid metabolites decreased in the control group and increased in the testosterone group. The linear mixed model revealed multiple significant interactions with androgenic steroid metabolites, and these were higher (p < 0.05) in the testosterone group compared to the placebo group during severe energy deficit (Fig. 4, Supplement 1). This trend was generally present except for several estrogenic DHEA metabolites (i.e., adrostendediol (3α, 17α) monosulfate (2)), which were lower (p < 0.05) in the testosterone group during severe energy deficit compared to the placebo group (Fig. 4, Supplement 1). However, the changes reversed during Phase 3 when testosterone was not being administered, and androgenic steroid metabolites were lower (p < 0.05) in the testosterone group compared to placebo (Fig. 4, Supplement 1).

In general, serum fatty acid metabolites increased in response to severe energy deficit and decreased during the recovery period in both treatment groups (Fig. 4). Compared to CON, fatty acid metabolites were higher during Phase 2 and lower during Phase 3 in the testosterone group; specifically, long-chain acylcarnitines, unsaturated fatty acids, and medium chain fatty acids (Fig. 4, Supplement 3). Long-chain acylcarnitines and dicarboxylated acylcarnitines were consistently higher (p < 0.05) in the testosterone group at SED14 and SED28 compared to the placebo group (Fig. 4, Supplement 1). Additionally, the testosterone group had lower (p < 0.05) levels of acylcarnitines, unsaturated fatty acids, and medium chain fatty acids at WR compared to the placebo group (Fig. 4, Supplement 1).

Amino acid metabolites generally decreased during severe energy deficit and increased during recovery in both treatments (Fig. 4, Supplement 4). However, compared to the placebo group, phenylalanine, tryptophan, leucine, isoleucine, and valine metabolites were lower (p < 0.05) in the testosterone group during severe energy deficit (Fig. 4, Supplement 1) and higher during recovery (i.e., Phase 3). Additionally, urea cycle (Fig. 4) and creatine metabolites (Supplement 1) were higher (p < 0.05) in the testosterone group during FL and WR compared to the placebo group.

**Fig. 5:**
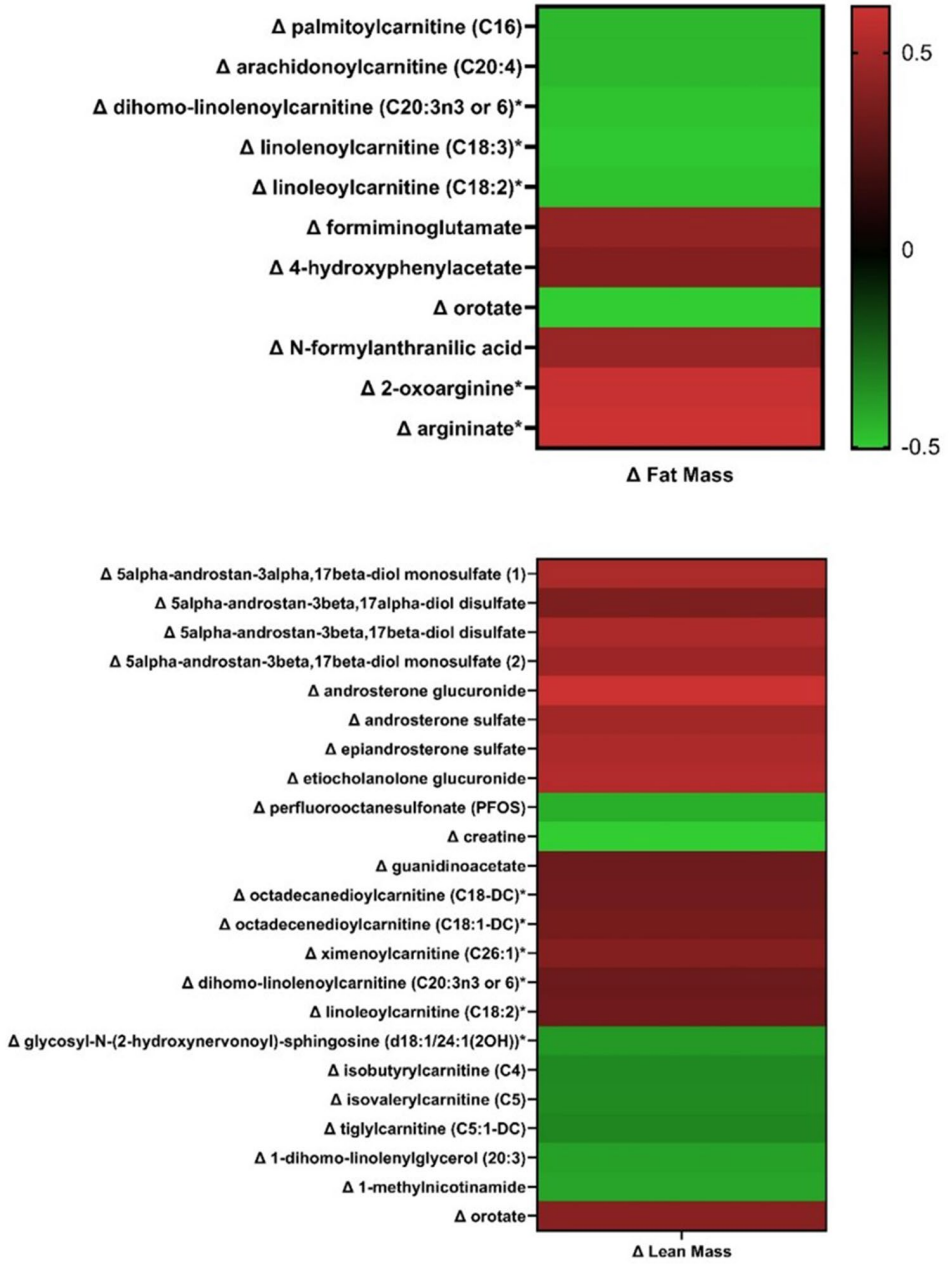
Heatmap of Pearson’s correlation coefficients between changes in analytes and changes in fat mass (top) and lean mass (bottom) after severe energy-deficit

Changes in lean mass were associated (FDR < 0.05) with changes in 23 analytes (Fig. 5, Supplement 5). Pearson’s product-moment correlations indicated that changes in lean mass were positively correlated with changes in several metabolites of androgenic steroid metabolism (N = 8, r = 0.42 to 0.70) and with multiple acylcarnitines (N = 5; r = 0.37 to 0.44). In contrast, changes in lean mass were inversely related to changes in metabolites of leucine, isoleucine and valine metabolism (N = 3, r = − 0.36 to − 0.37). Changes in fat mass were associated with changes in 11 metabolites (Fig. 5, Supplement 6). Changes in fat mass was inversely related to changes in acylcarnitines (N = 5, r = − 0.46 to − 0.49) and positively associated with changes in urea cycle, arginine, and proline metabolites (N = 2, r = 0.60 to 0.62). The changes in androgenic steroids were moderately-to-strongly correlated to the changes in acylcarnitines (N = 4, r = − 0.48 to 0.56, Supplement 7).

## Discussion

This study assessed the effects of testosterone administration on serum metabolomic signatures and determined if metabolomic changes were related to changes in body composition in healthy men during severe energy deficit. The major findings were that testosterone-treated volunteers had higher androgenic steroid (i.e., 5alpha-androstan-3alpha,17beta-diol monosulfate (1), androsterone sulfate) and fatty acid metabolites (acylcarnitine, unsaturated fatty acids, medium chain fatty acids) and lower amino acid metabolites during severe energy deficit (i.e., Phase 2) compared to the placebo group. During the recovery period (i.e., Phase 3), when testosterone was not administered and the severe energy deficit had ended, many of these changes reversed. Additionally, changes in lean mass during severe energy deficit were related to changes in androgenic steroids and acylcarnitines, and inversely related to changes in amino acid metabolites. Thus, these data suggest doses of testosterone enanthate (200 mg/wk in sesame oil), administered weekly during severe energy deficit altered serum metabolomic signatures in a manner corresponding to increases in lean mass.

To our knowledge, this is the first study to evaluate the effects of testosterone during severe energy deficit on the serum metabolome. Testosterone inhibits lipid uptake and lipoprotein-lipase activity in adipocytes, and upregulates lipolytic β-adrenergic receptors (De Pergola, 2000) resulting in reductions in fat mass. This study also found that testosterone administration perturbed fatty acid oxidation. Increases in acylcarnitines, a subclass of lipids, were found which corroborates the findings of Monnerat et al. (2018) who reported increased acylcarnitines in adult Wistar rats after 30 d of testosterone administration (1 mg/kg/day). These findings are also in agreement with Guedes et al. (2022) who reported that acylcarnitine levels change in a testosterone-sensitive direction in humans after pharmacological castration and subsequent testosterone administration. Testosterone mediates the upregulation in long chain fatty acid oxidation of hexadecoanoic acid in myotubes (Salehzadeh et al., 2011) and can increase acylcarnitines in conjunction with reductions in body fat. Previously, we reported that the placebo group lost more body mass during the severe energy deficit phase of this study (Pasiakos et al., 2019). Since an equivalent energy deficit was imposed on both groups, it is possible that testosterone administration led to mobilization/utilization of lipid substrates for energy provision, rather than less energy dense protein stores. In support of this, we found that acylcarnitines were inversely correlated to fat mass and positively related to lean mass after severe energy deficit.

As part of this clinical trial, our laboratory reported that testosterone administration during severe energy deficit upregulated skeletal muscle androgen receptors and translation capacity, and attenuated proteolysis (Howard et al., 2020). Here we show that serum amino acids were lower in the testosterone group during severe energy deficit compared to the control group, which likely indicates lower muscle protein breakdown. Androgens seem to play a vital role in shifting muscle protein balance in favor of protein accretion (Rossetti et al., 2017; Sheffield-Moore, 2000). In this investigation, when we administered a slow-release form of testosterone for 28 days, muscle proteolysis was attenuated and lean mass increased. In support of these findings, we report here the changes in lean mass observed were inversely related to amino acid metabolite levels. These findings are consistent with other investigations that found hypogonadism is associated with elevated proteolysis (Jiao et al., 2009) and high adiposity (Antonic et al., 2020). Again, this supports that testosterone administration may improve body composition during severe energy deficit by altering substrate utilization by upregulating fatty acid oxidation and downregulating proteolytic activity from skeletal muscle.

This study also found that many of the metabolomic changes observed due to severe energy deficit in volunteers treated with testosterone were reversed after its administration ended. Cessation of testosterone administration led to an increase in leucine (isovalerylcarnitine (C5)) and valine (isobutyrylcarnitine (C4)) catabolites. Valine and leucine are metabolized by branched-chain amino transferase (BCAT) to generate branched-chain ketoacids and glutamate. This reaction also is catalyzed by vitamin B6 metabolites, which were also elevated in the testosterone group during the recovery period. The BCAT reaction produces glutamate which donates its amino group to urea cycle metabolites. Thus, the increases in branched chain amino acid metabolites may explain the increases in the urea cycle and creatine metabolites in the testosterone group during the recovery period. Urea and creatine metabolites are markers of renal function, which testosterone is believed to influence in a dose-dependent fashion (Hajjar et al., 1997; Yassin et al., 2020). The observed increases in urea cycle and creatine metabolites in our study could be indicating that renal function is decreased after testosterone cessation. Alternatively, these findings could be a function of returning to basal muscle protein breakdown. Additionally, most of the fatty acid metabolites (i.e., decanoylcarnitine (C10), laurate) that were higher during Phase 2 decreased after testosterone was no longer administered. A similar trend was seen for androgenic steroid metabolites. Androgenic steroids increased during severe energy deficit and decreased during recovery (i.e., 5alpha-androstan-3alpha, 17beta-diol disulfate, 5alpha-androstan-3beta, 17beta-diol monosulfate (2)) in the testosterone group. This suggests that testosterone administration played a significant role in mediating both the amino acid and lipid signatures of healthy men during severe energy deficit, and that these changes are reversed upon return to normocaloric conditions without supplemental testosterone. This study also highlighted that the metabolomic changes observed were associated with improvements in body composition during Phase 2. Thus, it is possible that the improvements in body composition observed may also reverse in conjunction with changes in the serum metabolome after cessation of testosterone administration. A potential avenue for future research is to determine if the relationship between the metabolome and body composition remains when employing chronic or cyclical administration of testosterone.

The PCA analysis we conducted demonstrated some separation of the metabolome by treatment condition after 14 days of severe energy deficit but orthogonal PLS-DA indicated that the between-group differences in the global metabolome after 14 days was not significant. Alternatively, univariate testing revealed many between group metabolic differences. These between-group differences were primarily due to higher acylcarnitines (e.g., myristoylcarnitine (C14), palmitoylcarnitine (C16)) and lower amino acid metabolites (e.g., isovalerate, 4-hydroxyphenylacetate, xanthurenate) at SED14 in the testosterone group. Again, these differences likely reflect testosterone’s effect on inhibiting lipid uptake/lipolytic activity and attenuated proteolysis. However, testosterone’s metabolic effects did not persist throughout Phase 2, which could indicate that the severe energy deficit imposed eventually outweighed the metabolic effects of testosterone. This suggests the dose and timing of testosterone administration used in this study may not be the optimal intervention for the prolonged severe energy deficits seen in military personnel (Tharion et al., 2005). However, other formulations of testosterone may be more effective.

The strengths of this study include its between-subjects, double-blind, randomized, placebo-controlled design, 100% retention of participants after randomization, and ≥ 89% adherence to the severe energy deficit protocol. There were limitations as well. The 200 mg testosterone dose was intended to attenuate declines in circulating testosterone previously observed during severe energy deficit. However, these decrements were not as severe as hypothesized leading to testosterone concentrations that were higher than basal concentrations in the testosterone-treated group. It has been previously established that hypogonadal patients with type 2 diabetes improve glycaemic control with testosterone replacement therapy (Kapoor et al., 2006). It is possible that severe energy deficit did not produce hypogonadism to the same extent and decreased our sensitivity to detect differences in glucose/carbohydrate metabolism between the treatment groups. In this study, severe energy deficit was primarily induced by increasing exercise-induced energy expenditure. Thus, our findings may not generalize to other models of severe hypocaloric feeding.

## Conclusion

Testosterone administration during exercise- and diet-induced severe energy deficit, and weight regain due to refeeding significantly influenced serum metabolomic signatures. Testosterone administration resulted in higher levels of androgenic steroid (i.e., 5alpha-androstan-3alpha, 17beta-diol monosulfate (Berryman et al., 2018), androsterone sulfate) and fatty acid metabolites (acylcarnitine, unsaturated fatty acids, medium chain fatty acids) and lower levels of amino acids metabolites (i.e., isovalerate [i5:0], isovalerylcarnitine [C5]) during severe energy deficit. However, these changes were transient and reversed during the recovery period when testosterone was not administered and energy deficit ended. In addition, testosterone administration led to improvements in body composition during severe energy deficit, which were associated with changes in serum metabolomic signatures. These findings suggest testosterone administration may: (1) alter substrate utilization such that lipid substrates are preferentially utilized over amino acids; and (2) these metabolomic changes may be associated with attenuated muscle proteolysis. However, we also noted these changes are transient and are reversed after cessation of testosterone administration. Thus, it is possible that the body composition changes observed are also transient and future investigations are needed to ascertain the metabolic implications of chronic or cyclical testosterone administration. These findings are important for individuals such as military personnel, woodland firefighters, and competitive athletes who encounter severe energy deficits when muscle function and performance are essential for the successful execution of the mission or for performance of their sport.

## Supplementary Information

Below is the link to the electronic supplementary material.

Supplementary file1 (DOCX 36 KB)

Supplementary file2 (PNG 98 KB)

Supplementary file3 (PNG 158 KB)

Supplementary file4 (PNG 174 KB)

Supplementary file5 (JPG 6994 KB)

Supplementary file6 (JPG 5984 KB)

Supplementary file7 (DOCX 15 KB)

Supplementary file8 (DOCX 18 KB)

## Data Availability

Supplementary data are available via Figshare at https://figshare.com/s/2e896b1bcacdb1d6b300.
