# Supplementary material for: Metabolomics of testosterone enanthate administration during severe-energy deficit
Source: Metabolomics. 2022 Nov 30;18(12):100. doi: 10.1007/s11306-022-01955-y (PMC9712311; doi:10.1007/s11306-022-01955-y)
Supplement: Supplementary file 1 — Supplementary file1 (DOCX 36 KB) [file 11306_2022_1955_MOESM1_ESM.docx]

Stein et al. “Metabolomics of testosterone enanthate administration during severe-energy deficit”

*Metabolomics*; Corresponding author: Harris R. Lieberman, harris.r.lieberman.civ@mail.mil

| **Supplementary Table 1:** Serum Metabolomic Differences Between Testosterone and Placebo Treatments | | | | | | |
| --- | --- | --- | --- | --- | --- | --- |
|  | CON | SED14 | SED28 | FL | WR | q-value |
| Acetylated Peptides: phenylacetylcarnitine | − | − | − | − | ↑*** | 0.045 |
| Androgenic Steroids: 5alpha-androstan-3alpha,17alpha-diol monosulfate | − | − | − | ↓* | ↓*** | 0.000 |
| Androgenic Steroids: 5alpha-androstan-3alpha,17beta-diol 17-glucuronide | − | ↑**** | ↑**** | − | − | 0.000 |
| Androgenic Steroids: 5alpha-androstan-3alpha,17beta-diol disulfate | − | − | ↑** | ↓**** | ↓**** | 0.000 |
| Androgenic Steroids: 5alpha-androstan-3alpha,17beta-diol monosulfate (1) | − | ↑**** | ↑**** | − | − | 0.000 |
| Androgenic Steroids: 5alpha-androstan-3alpha,17beta-diol monosulfate (2) | − | − | − | ↓**** | ↓**** | 0.000 |
| Androgenic Steroids: 5alpha-androstan-3beta,17alpha-diol disulfate | − | ↑* | ↑* | − | ↓* | 0.000 |
| Androgenic Steroids: 5alpha-androstan-3beta,17beta-diol disulfate | − | ↑**** | ↑**** | − | − | 0.000 |
| Androgenic Steroids: 5alpha-androstan-3beta,17beta-diol monosulfate (2) | − | ↑**** | ↑**** | − | − | 0.000 |
| Androgenic Steroids: androstenediol (3alpha, 17alpha) monosulfate (2) | − | ↓* | ↓**** | ↓*** | ↓* | 0.000 |
| Androgenic Steroids: androstenediol (3alpha, 17alpha) monosulfate (3) | − | ↓* | ↓** | ↓* | − | 0.014 |
| Androgenic Steroids: androsterone glucuronide | − | − | − | − | ↑*** | 0.000 |
| Androgenic Steroids: androsterone sulfate | − | ↑**** | ↑**** | − | − | 0.000 |
| Androgenic Steroids: epiandrosterone sulfate | − | ↑**** | ↑**** | − | − | 0.000 |
| Androgenic Steroids: etiocholanolone glucuronide | − | ↑* | ↑** | − | − | 0.000 |
| Benzoate Metabolism: o-cresol sulfate | − | − | − | − | ↑** | 0.000 |
| Ceramides: ceramide (d18:2/24:1, d18:1/24:2)* | − | ↑** | − | − | − | 0.004 |
| Chemical: 2-naphthol sulfate | − | − | − | − | ↑* | 0.050 |
| Chemical: 3-hydroxypyridine sulfate | − | − | − | − | ↑* | 0.000 |
| Chemical: 6-hydroxyindole sulfate | − | ↓** | − | − | ↑* | 0.035 |
| Chemical: perfluorooctanesulfonate (PFOS) | − | − | − | − | − | 0.002 |
| Creatine Metabolism: creatine | − | − | − | ↑**** | ↑**** | 0.024 |
| Creatine Metabolism: guanidinoacetate | − | − | ↑** | − | − | 0.000 |
| Fatty Acid Metabolism (Acyl Carnitine, Dicarboxylate): octadecanedioylcarnitine (C18-DC)* | − | ↑*** | ↑* | − | − | 0.009 |
| Fatty Acid Metabolism (Acyl Carnitine, Dicarboxylate): octadecenedioylcarnitine (C18:1-DC)* | − | ↑*** | ↑* | − | − | 0.041 |
| Fatty Acid Metabolism (Acyl Carnitine, Long Chain Saturated): cerotoylcarnitine (C26)* | − | ↑* | ↑*** | ↓* | ↓**** | 0.029 |
| Fatty Acid Metabolism (Acyl Carnitine, Long Chain Saturated): myristoylcarnitine (C14) | − | ↑* | − | − | ↓* | 0.000 |
| Fatty Acid Metabolism (Acyl Carnitine, Long Chain Saturated): palmitoylcarnitine (C16) | − | ↑* | − | − | − | 0.004 |
| Fatty Acid Metabolism (Acyl Carnitine, Long Chain Saturated): stearoylcarnitine (C18) | − | ↑* | ↑* | − | − | 0.005 |
| Fatty Acid Metabolism (Acyl Carnitine, Medium Chain): decanoylcarnitine (C10) | − | − | − | − | ↓*** | 0.015 |
| Fatty Acid Metabolism (Acyl Carnitine, Medium Chain): laurylcarnitine (C12) | − | − | − | − | ↓** | 0.015 |
| Fatty Acid Metabolism (Acyl Carnitine, Monounsaturated): nervonoylcarnitine (C24:1)* | − | ↑** | ↑* | − | ↓** | 0.026 |
| Fatty Acid Metabolism (Acyl Carnitine, Monounsaturated): oleoylcarnitine (C18:1) | − | ↑* | ↑** | − | ↓** | 0.000 |
| Fatty Acid Metabolism (Acyl Carnitine, Monounsaturated): palmitoleoylcarnitine (C16:1)* | − | ↑* | − | − | ↓* | 0.000 |
| Fatty Acid Metabolism (Acyl Carnitine, Monounsaturated): ximenoylcarnitine (C26:1)* | − | ↑**** | ↑**** | − | − | 0.018 |
| Fatty Acid Metabolism (Acyl Carnitine, Polyunsaturated): adrenoylcarnitine (C22:4)* | − | − | − | − | ↓*** | 0.000 |
| Fatty Acid Metabolism (Acyl Carnitine, Polyunsaturated): arachidonoylcarnitine (C20:4) | − | − | ↑* | − | − | 0.020 |
| Fatty Acid Metabolism (Acyl Carnitine, Polyunsaturated): dihomo-linolenoylcarnitine (C20:3n3 or 6)* | − | ↑**** | ↑**** | ↓* | − | 0.011 |
| Fatty Acid Metabolism (Acyl Carnitine, Polyunsaturated): linolenoylcarnitine (C18:3)* | − | ↑* | ↑* | − | − | 0.000 |
| Fatty Acid Metabolism (Acyl Carnitine, Polyunsaturated): linoleoylcarnitine (C18:2)* | − | ↑*** | ↑**** | − | − | 0.026 |
| Fatty Acid, Branched: (14 or 15)-methylpalmitate (a17:0 or i17:0) | − | − | − | − | ↓*** | 0.013 |
| Fatty Acid, Branched: (16 or 17)-methylstearate (a19:0 or i19:0) | − | − | − | ↓* | ↓**** | 0.013 |
| Fatty Acid, Branched: pristanate | − | ↑* | ↑* | − | ↓* | 0.001 |
| Food Component/Plant: indolin-2-one | − | − | − | − | ↑** | 0.033 |
| Food Component/Plant: phytanate | − | − | − | − | ↓* | 0.022 |
| Gamma-glutamyl Amino Acid: gamma-glutamyltyrosine | − | − | − | − | ↑* | 0.012 |
| Glycine, Serine and Threonine Metabolism: sarcosine | − | − | − | ↑*** | ↑**** | 0.001 |
| Hemoglobin and Porphyrin Metabolism: biliverdin | − | ↑** | − | ↑* | − | 0.000 |
| Hexosylceramides (HCER): glycosyl-N-(2-hydroxynervonoyl)-sphingosine (d18:1/24:1(2OH))* | − | − | ↓** | − | − | 0.005 |
| Histidine Metabolism: 4-imidazoleacetate | − | − | ↑*** | − | − | 0.007 |
| Histidine Metabolism: formiminoglutamate | − | − | − | ↑**** | ↑**** | 0.035 |
| Leucine, Isoleucine and Valine Metabolism: 2-methylbutyrylcarnitine (C5) | − | − | − | − | − | 0.000 |
| Leucine, Isoleucine and Valine Metabolism: isobutyrylcarnitine (C4) | − | − | − | − | ↑**** | 0.039 |
| Leucine, Isoleucine and Valine Metabolism: isovalerate (i5:0) | − | ↓**** | − | − | − | 0.000 |
| Leucine, Isoleucine and Valine Metabolism: isovalerylglycine | − | − | − | − | ↑*** | 0.001 |
| Leucine, Isoleucine and Valine Metabolism: N-acetylisoleucine | − | − | − | − | ↑* | 0.000 |
| Leucine, Isoleucine and Valine Metabolism: tiglylcarnitine (C5:1-DC) | − | − | − | ↑**** | ↑**** | 0.007 |
| Leucine, Isoleucine, and Valine Metabolism: isovalerylcarnitine (C5) | − |  | ↓** | ↑* | ↑*** | 0.033 |
| Long Chain Monounsaturated Fatty Acid: 10-nonadecenoate (19:1n9) | − | − | − | − | ↓**** | 0.000 |
| Long Chain Monounsaturated Fatty Acid: myristoleate (14:1n5) | − | − | − | − | ↓**** | 0.013 |
| Long Chain Polyunsaturated Fatty Acid (n3 and n6): docosahexaenoate (DHA; 22:6n3) | − | − | − | − | ↓**** | 0.041 |
| Long Chain Polyunsaturated Fatty Acid (n3 and n6): docosapentaenoate (n3 DPA; 22:5n3) | − | − | − | − | ↓**** | 0.005 |
| Long Chain Polyunsaturated Fatty Acid (n3 and n6): docosapentaenoate (n6 DPA; 22:5n6) | − | − | − | − | ↓*** | 0.033 |
| Long Chain Saturated Fatty Acid: nonadecanoate (19:0) | − | − | − | − | ↓* | 0.033 |
| Lysine Metabolism: 2-aminoadipate | − | − | − | − | ↑* | 0.034 |
| Lysine Metabolism: 6-oxopiperidine-2-carboxylate | − | − | − | − | ↑*** | 0.033 |
| Lysine Metabolism: glutarylcarnitine (C5-DC) | − | − | − | ↑* | − | 0.022 |
| Lysophospholipid: 1-palmitoyl-GPE (16:0) | ↑** | ↑**** | ↑* | − | − | 0.041 |
| Medium Chain Fatty Acid: caprate (10:0) | − | − | − | ↓* | ↓**** | 0.026 |
| Medium Chain Fatty Acid: laurate (12:0) | − | − | − | − | ↓**** | 0.041 |
| Methionine, Cysteine, SAM and Taurine Metabolism: methionine sulfone | − | − | − | ↑* | − | 0.013 |
| Methionine, Cysteine, SAM and Taurine Metabolism: S-methylcysteine | − | − | ↑* | − | − | 0.044 |
| Monoacylglycerol: 1-dihomo-linolenylglycerol (20:3) | − | − | − | ↓** | − | 0.028 |
| Nicotinate and Nicotinamide Metabolism: 1-methylnicotinamide | − | ↓* | ↓*** | ↓**** | − | 0.039 |
| Nicotinate and Nicotinamide Metabolism: N1-Methyl-2-pyridone-5-carboxamide | − | − | − | ↑**** | − | 0.000 |
| Nicotinate and Nicotinamide Metabolism: N1-Methyl-4-pyridone-3-carboxamide | − | − | − | ↑**** | ↑* | 0.007 |
| Nicotinate and Nicotinamide Metabolism: quinolinate | − | − | − | ↑* | − | 0.014 |
| Partially Characterized Molecules: glutamine_degradant* | − | − | − | − | − | 0.001 |
| Pentose Metabolism: ribonate | − | − | − | − | − | 0.039 |
| Phenylalanine Metabolism: 2-hydroxyphenylacetate | − | − | − | − | − | 0.041 |
| Phenylalanine Metabolism: 4-hydroxyphenylacetate | − | ↓**** | − | − | − | 0.033 |
| Phenylalanine Metabolism: N-acetylphenylalanine | − | − | − | ↑*** | ↑**** | 0.009 |
| Phenylalanine Metabolism: phenyllactate (PLA) | − | ↓*** | ↓* | − | − | 0.011 |
| Phenylalanine Metabolism: phenylpyruvate | − | − | − | − | ↑* | 0.000 |
| Phosphatidylethanolamine (PE): 1-oleoyl-2-docosahexaenoyl-GPE (18:1/22:6)* | − | ↓* | − | − | − | 0.037 |
| Phospholipid Metabolism: glycerophosphoinositol* | − | ↓** | − | − | ↓* | 0.023 |
| Polyamine Metabolism: spermidine | − | ↑** | − | ↑* | ↓* | 0.019 |
| Primary Bile Acid Metabolism: glycochenodeoxycholate glucuronide (1) | − | − | ↑** | − | − | 0.020 |
| Pyrimidine Metabolism, Orotate containing: orotate | − | ↑* | ↑** | − | − | 0.041 |
| Tryptophan Metabolism: 3-indoxyl sulfate | − | ↓** | − | − | ↑* | 0.000 |
| Tryptophan Metabolism: indoleacetate | − | − | − | − | ↑* | 0.001 |
| Tryptophan Metabolism: kynurenate | − | − | − | ↑**** | ↑** | 0.043 |
| Tryptophan Metabolism: N-formylanthranilic acid | − | − | − | − | − | 0.000 |
| Tryptophan Metabolism: picolinate | − | − | − | ↑*** | ↑**** | 0.000 |
| Tryptophan Metabolism: xanthurenate | − | ↓* | − | ↑** | ↑** | 0.001 |
| Tyrosine Metabolism: 3-(4-hydroxyphenyl)lactate | − | − | − | − | − | 0.000 |
| Urea cycle; Arginine and Proline Metabolism: 2-oxoarginine* | − | − | − | ↑** | ↑**** | 0.033 |
| Urea cycle; Arginine and Proline Metabolism: argininate* | − | − | − | ↑**** | ↑**** | 0.000 |
| Urea cycle; Arginine and Proline Metabolism: homoarginine | − | − | − | ↑* | − | 0.000 |
| Urea cycle; Arginine and Proline Metabolism: N-acetylarginine | − | − | − | − | − | 0.000 |
| Urea cycle; Arginine and Proline Metabolism: N-acetylcitrulline | − | − | − | − | − | 0.000 |
| Vitamin B6 Metabolism: pyridoxate | − | − | − | ↑*** | ↑**** | 0.029 |
| Bonferroni adjusted p-values: * < 0.05. ** < 0.01, *** < 0.005, **** < 0.001 | | | | | | |
| ↑ Higher than control | | | | | | |
| ↓ Lower than control | | | | | | |
| − Not different from control | | | | | | |
