# Supplementary figures and images for: Metabolomics of testosterone enanthate administration during severe-energy deficit
Source: Metabolomics. 2022 Nov 30;18(12):100. doi: 10.1007/s11306-022-01955-y (PMC9712311; doi:10.1007/s11306-022-01955-y)

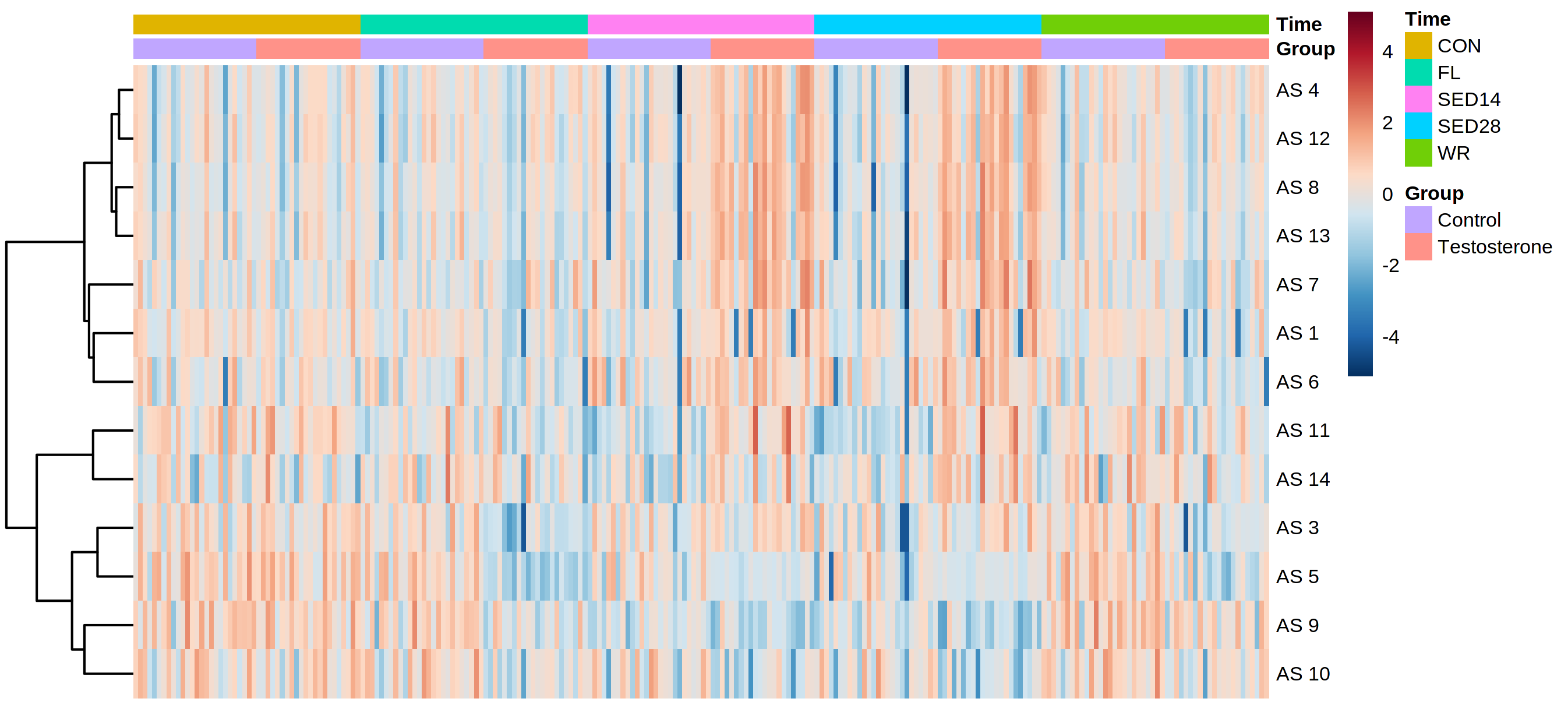

Supplement: Supplementary file 2 — Supplementary file2 (PNG 98 KB) [file 11306_2022_1955_MOESM2_ESM.png]

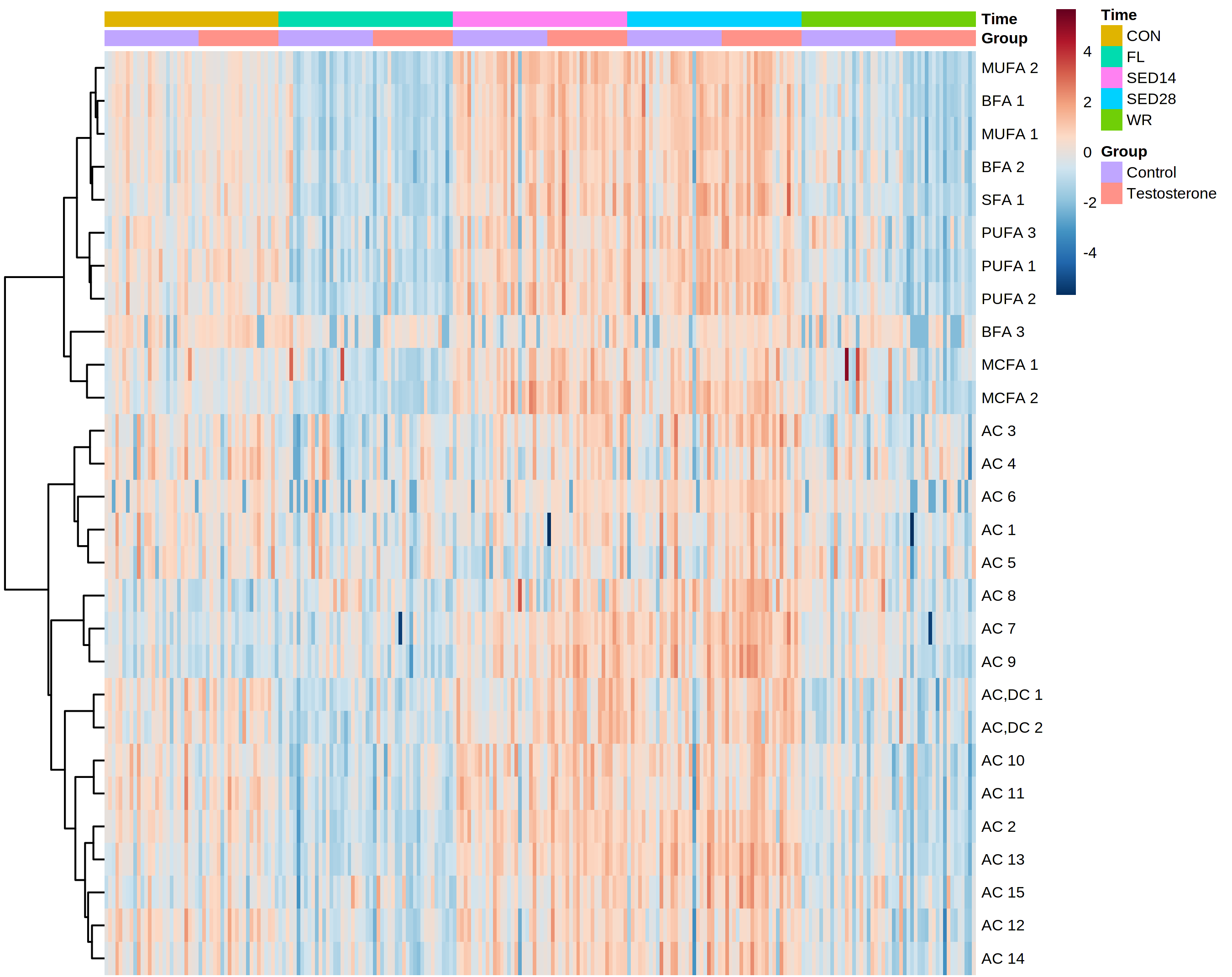

Supplement: Supplementary file 3 — Supplementary file3 (PNG 158 KB) [file 11306_2022_1955_MOESM3_ESM.png]

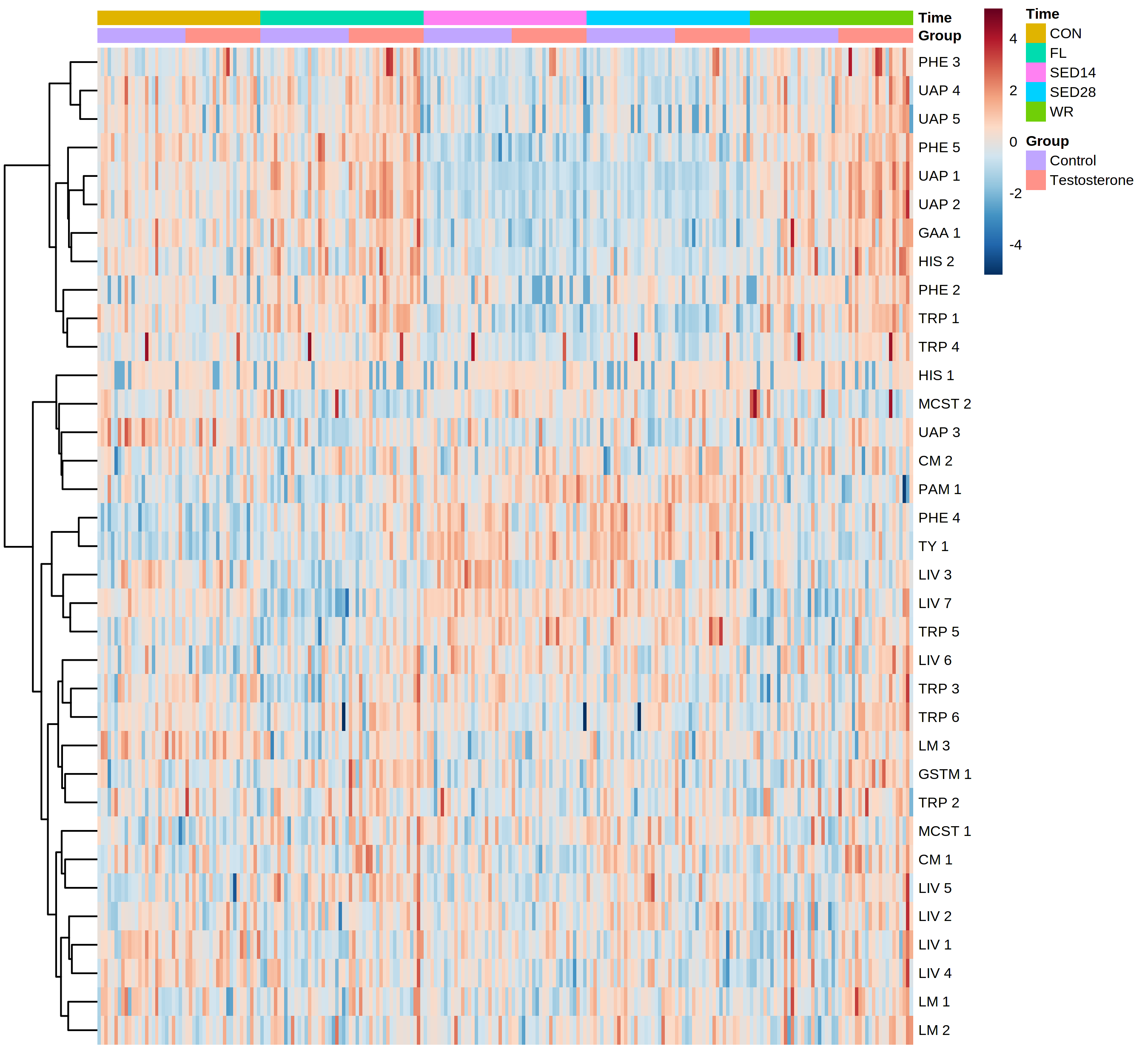

Supplement: Supplementary file 4 — Supplementary file4 (PNG 174 KB) [file 11306_2022_1955_MOESM4_ESM.png]
