# Supplementary material for: Metabolomics of testosterone enanthate administration during severe-energy deficit
Source: Metabolomics. 2022 Nov 30;18(12):100. doi: 10.1007/s11306-022-01955-y (PMC9712311; doi:10.1007/s11306-022-01955-y)
Supplement: Supplementary file 7 — Supplementary file7 (DOCX 15 KB) [file 11306_2022_1955_MOESM7_ESM.docx]

Stein et al. “Metabolomics of testosterone enanthate administration during severe-energy deficit”

| **Supplement 7:** Relationship Between Androgenic Steroid Metabolites and Changes in the Serum Metabolome | | | | |
| --- | --- | --- | --- | --- |
| **Metabolite** | **Androgen steroid metabolites** | **p-value** | **q-value** | **Pearson's r** |
| Δ adrenoylcarnitine (C22:4) | Δ 5alpha-androstan-3beta,17beta-diol disulfate | 0.000 | 0.017 | 0.503 |
| Δ adrenoylcarnitine (C22:4) | Δ 5alpha-androstan-3alpha,17beta-diol monosulfate (2) | 0.000 | 0.008 | 0.563 |
| Δ cerotoylcarnitine (C26) | Δ androsterone glucuronide | 0.001 | 0.031 | 0.470 |
| Δ cerotoylcarnitine (C26) | Δ androsterone sulfate | 0.001 | 0.046 | 0.446 |
| Δ N-acetylisoleucine | Δ 5alpha-androstan-3beta,17alpha-diol disulfate | 0.001 | 0.042 | 0.457 |
| Δ nervonoylcarnitine (C24:1) | Δ androstenediol (3alpha, 17alpha) monosulfate (2) | 0.000 | 0.027 | -0.481 |
| Δ oleoylcarnitine (C18:1) | Δ androsterone glucuronide | 0.001 | 0.044 | 0.453 |
| Δ oleoylcarnitine (C18:1) | Δ 5alpha-androstan-3alpha,17beta-diol monosulfate (1) | 0.000 | 0.008 | 0.551 |
| Δ oleoylcarnitine (C18:1) | Δ androsterone sulfate | 0.000 | 0.008 | 0.546 |
| Δ oleoylcarnitine (C18:1) | Δ epiandrosterone sulfate | 0.000 | 0.008 | 0.420 |
| Δ oleoylcarnitine (C18:1) | Δ 5alpha-androstan-3beta,17alpha-diol disulfate | 0.000 | 0.017 | 0.510 |
| Δ oleoylcarnitine (C18:1) | Δ 5alpha-androstan-3beta,17beta-diol monosulfate (2) | 0.000 | 0.017 | 0.508 |
| Δ oleoylcarnitine (C18:1) | Δ 5alpha-androstan-3beta,17beta-diol disulfate | 0.000 | 0.027 | 0.481 |
| Δ stearoylcarnitine (C18) | Δ 5alpha-androstan-3alpha,17beta-diol monosulfate (1) | 0.000 | 0.027 | 0.478 |
| Δ stearoylcarnitine (C18) | Δ 5alpha-androstan-3beta,17beta-diol monosulfate (2) | 0.001 | 0.046 | 0.446 |
| Δ stearoylcarnitine (C18) | Δ androsterone sulfate | 0.001 | 0.048 | 0.443 |
| q-value represents Benjamoni-hochberg false discovery rate correction. Change scores reflect the difference between severe energy deficit and free-living, controlled eating. | | | | |

Metabolomics; Corresponding author: Harris R. Lieberman, harris.r.lieberman.civ@mail.mil
