# Supplementary material for: Metabolomics of testosterone enanthate administration during severe-energy deficit
Source: Metabolomics. 2022 Nov 30;18(12):100. doi: 10.1007/s11306-022-01955-y (PMC9712311; doi:10.1007/s11306-022-01955-y)
Supplement: Supplementary file 8 — Supplementary file8 (DOCX 18 KB) [file 11306_2022_1955_MOESM8_ESM.docx]

| **Abbreviation** | **Metabolite** |
| --- | --- |
| AC,DC 1 | cerotylcarnitine (C18:1-DC)* |
| AC,DC 2 | cerotylcarnitine (C18-DC)* |
| AC 1 | arachidonoylcarnitine (C20:4) |
| AC 2 | cerotylcarnitine (C16:1)* |
| AC 3 | cerotylcarnitine (C18:2)* |
| AC 4 | cerotylcarnitine (C18:3)* |
| AC 5 | cerotylcarnitine (C20:3n3 or 6)* |
| AC 6 | cerotylcarnitine (C22:4)* |
| AC 7 | cerotylcarnitine (C24:1)* |
| AC 8 | cerotylcarnitine (C26)* |
| AC 9 | cerotylcarnitine (C26:1)* |
| AC 10 | decanoylcarnitine (C10) |
| AC 11 | laurylcarnitine (C12) |
| AC 12 | myristoylcarnitine (C14) |
| AC 13 | oleoylcarnitine (C18:1) |
| AC 14 | palmitoylcarnitine (C16) |
| AC 15 | stearoylcarnitine (C18) |
| AP 1 | phenylacetylcarnitine |
| AS 1 | 5alpha-androstan-3alpha,17alpha-diol monosulfate |
| AS 2 | 5alpha-androstan-3alpha,17beta-diol 17-glucuronide |
| AS 3 | 5alpha-androstan-3alpha,17beta-diol disulfate |
| AS 4 | 5alpha-androstan-3alpha,17beta-diol monosulfate (1) |
| AS 5 | 5alpha-androstan-3alpha,17beta-diol monosulfate (2) |
| AS 6 | 5alpha-androstan-3beta,17alpha-diol disulfate |
| AS 7 | 5alpha-androstan-3beta,17beta-diol disulfate |
| AS 8 | 5alpha-androstan-3beta,17beta-diol monosulfate (2) |
| AS 9 | androstenediol (3alpha, 17alpha) monosulfate (2) |
| AS 10 | androstenediol (3alpha, 17alpha) monosulfate (3) |
| AS 11 | androsterone glucuronide |
| AS 12 | androsterone sulfate |
| AS 13 | epiandrosterone sulfate |
| AS 14 | etiocholanolone glucuronide |
| BFA 1 | (14 or 15)-methylpalmitate (a17:0 or i17:0) |
| BFA 2 | (16 or 17)-methylstearate (a19:0 or i19:0) |
| BFA 3 | pristanate |
| BM 1 | o-cresol sulfate |
| CA 1 | ceramide (d18:2/24:1, d18:1/24:2)* |
| CH 1 | 2-naphthol sulfate |
| CH 2 | 3-hydroxypyridine sulfate |
| CH 3 | 6-hydroxyindole sulfate |
| CH 4 | perfluorooctanesulfonate (PFOS) |
| CM 1 | creatine |
| CM 2 | guanidinoacetate |
| FCP 1 | indolin-2-one |
| FCP 2 | phytanate |
| GAA 1 | gamma-glutamyltyrosine |
| GSTM 1 | sarcosine |
| HCER 1 | cerotylcarnitine (d18:1/24:1(2OH))* |
| HIS 1 | 4-imidazoleacetate |
| HIS 2 | formiminoglutamate |
| HPM 1 | biliverdin |
| LIV 1 | 2-methylbutyrylcarnitine (C5) |
| LIV 2 | isobutyrylcarnitine (C4) |
| LIV 3 | isovalerate (i5:0) |
| LIV 4 | isovalerylcarnitine (C5) |
| LIV 5 | isovalerylglycine |
| LIV 6 | N-acetylisoleucine |
| LIV 7 | tiglylcarnitine (C5:1-DC) |
| LM 1 | 2-aminoadipate |
| LM 2 | 6-oxopiperidine-2-carboxylate |
| LM 3 | glutarylcarnitine (C5-DC) |
| LP 1 | 1-palmitoyl-GPE (16:0) |
| MCFA 1 | caprate (10:0) |
| MCFA 2 | laurate (12:0) |
| MCST 1 | methionine sulfone |
| MCST 2 | S-methylcysteine |
| MG 1 | 1-dihomo-linolenylglycerol (20:3) |
| MUFA 1 | 10-nonadecenoate (19:1n9) |
| MUFA 2 | myristoleate (14:1n5) |
| NNM 1 | 1-methylnicotinamide |
| NNM 2 | N1-Methyl-2-pyridone-5-carboxamide |
| NNM 3 | N1-Methyl-4-pyridone-3-carboxamide |
| NNM 4 | quinolinate |
| PAM 1 | spermidine |
| PBAM 1 | glycochenodeoxycholate glucuronide (1) |
| PCM 1 | glutamine_degradant* |
| PE 1 | cerotylcarnitine (18:1/22:6)* |
| PHE 1 | 2-hydroxyphenylacetate |
| PHE 2 | 4-hydroxyphenylacetate |
| PHE 3 | N-acetylphenylalanine |
| PHE 4 | phenyllactate (PLA) |
| PHE 5 | phenylpyruvate |
| PLM 1 | glycerophosphoinositol* |
| PM 1 | ribonate |
| PN 1 | pyridoxate |
| PUFA 1 | docosahexaenoate (DHA; 22:6n3) |
| PUFA 2 | docosapentaenoate (n3 DPA; 22:5n3) |
| PUFA 3 | docosapentaenoate (n6 DPA; 22:5n6) |
| PYM 1 | orotate |
| SFA 1 | nonadecanoate (19:0) |
| TRP 1 | 3-indoxyl sulfate |
| TRP 2 | indoleacetate |
| TRP 3 | kynurenate |
| TRP 4 | N-formylanthranilic acid |
| TRP 5 | picolinate |
| TRP 6 | xanthurenate |
| TY 1 | 3-(4-hydroxyphenyl)lactate |
| UAP 1 | 2-oxoarginine* |
| UAP 2 | argininate* |
| UAP 3 | homoarginine |
| UAP 4 | N-acetylarginine |
| UAP 5 | N-acetylcitrulline |
